# Cervical cancer screening willingness and associated health beliefs among predominantly college-educated young women attending gynecology clinics in Shenzhen, China

**DOI:** 10.3389/fpubh.2026.1969898

**Published:** 2026-10-12

**Authors:** Hui Li, Yan Ke, Meihua Wu, Lin Shen, Nan Jiang, Honggui Wang, Mei Wang, Xingdi Qiu, Qiuxiang Wu, Huijun Bai, Yan Wang, Yutao Gao, Wenming Cao, Yang Zhang

**Affiliations:** 1 Department of Gynecology, Shenzhen Hospital of Integrated Traditional Chinese and Western Medicine, Shenzhen, Guangdong, China; 2 Department of Gynecology, Pingshan District Central Hospital of Shenzhen, Shenzhen, Guangdong, China; 3 Department of Obstetrics and Gynecology, Shenzhen University General Hospital, Shenzhen, Guangdong, China; 4 Department of Gynecology, Shenzhen Third People's Hospital, Shenzhen, Guangdong, China

**Keywords:** cervical cancer screening, China, cues to action, Health Belief Model, perceived barriers, screening willingness, young women

## Abstract

**Purpose:**

This study examined cervical cancer screening willingness and its associations with Health Belief Model (HBM) constructs among predominantly college-educated young women attending gynecology outpatient clinics in Shenzhen, China.

**Methods:**

A cross-sectional convenience-sampling survey was conducted (May 2023–May 2026) among 2,317 young women recruited from gynecology outpatient clinics in Shenzhen, China. The primary outcome was personal willingness to undergo screening (single binary item). Self-reported screening within three years was an exploratory secondary outcome. Thirty-three Likert items measured seven HBM domains. Seven HBM domains were treated as investigator-constructed summary scores and examined only exploratorily.

**Results:**

Most participants were aged 18–24 years (96.8%), unmarried (97.1%), and college-educated (95.5%). Screening willingness was reported by 88.3% and screening within the previous three years by 9.5%. Because these indicators capture different constructs (future willingness vs. previous screening experience) and eligibility was not assessed, their difference was not interpreted as a willingness–behavior gap. Cronbach’s α ranged 0.890–0.965. In adjusted models, perceived barriers were inversely associated with willingness (aOR = 0.454, 95% CI 0.375–0.550), while cues to action (aOR = 1.399, 95% CI 1.189–1.646), perceived severity (aOR = 1.257, 95% CI 1.038–1.523), perceived benefits (aOR = 1.194, 95% CI 1.017–1.402), and HPV vaccination (aOR = 1.576, 95% CI 1.161–2.137) were positively associated.

**Conclusion:**

Willingness to undergo screening was commonly reported, whereas prior screening experience was limited. Perceived barriers and cues to action were key correlates. Findings support guideline-based prevention communication but are cross-sectional and limited by clinic-based convenience sampling in Shenzhen, China; causal effects cannot be inferred.

## 1 Introduction

Cervical cancer (CC) remains a major public health concern for women worldwide, despite the availability of highly effective primary prevention through HPV vaccination and secondary prevention through screening and treatment of precancerous lesions (1). The public-health value of screening depends on more than the existence of services: women must recognize when screening is appropriate, understand what screening can and cannot prevent, trust the health system, and be able to overcome emotional, logistical, and financial barriers (2, 3). These requirements are particularly important when vaccination, outpatient care, and digital health information are delivered through different parts of the health system (4).

Young women represent a distinctive population in CC prevention (5). They are often at an age when reproductive-health knowledge, HPV vaccination decisions, health beliefs, and future screening expectations are being formed (6, 7). Nevertheless, current screening behavior among young women cannot be interpreted in the same way as screening behavior among older screening-eligible women (8). Many young women may not yet meet age- or guideline-based screening criteria, and the clinical relevance of screening may also depend on sexual history and individual risk factors (9). Marital status should not be used as a proxy for screening eligibility (10). Therefore, limited past screening in this population should not automatically be interpreted as poor adherence or inadequate health behavior. At the same time, a low level of prior screening does not imply that prevention education is unnecessary; it may identify an opportunity to establish accurate knowledge, appropriate expectations, and confidence in future guideline-concordant care (11).

In this study, we use the term screening willingness to refer to self-reported general willingness to undergo cervical cancer screening (CCS) in the future. This single-item measure should not be equated with a fully specified behavioral intention, an immediate inclination to act, or guideline-concordant screening behavior; no independent composite score was constructed (12).

The Health Belief Model provides a useful theoretical framework for understanding screening willingness (13–15). It proposes that preventive health behavior is shaped by perceived susceptibility, perceived severity, perceived benefits, perceived barriers, cues to action, self-efficacy, and health motivation (16). In the context of CC prevention, the model also provides a practical structure for health education: susceptibility and severity messages should be accurate and non-alarmist; benefits should explain early detection and timely management; barriers such as privacy, pain, cost, and navigation may need to be addressed, and cues to action may help connect clinic encounters with reliable digital information (17). This framework may help distinguish education that supports appropriate future screening from messaging that unintentionally promotes screening outside guideline recommendations (16).

This study aimed to examine personal willingness to undergo CCS among predominantly college-educated young women attending gynecology outpatient clinics in China. Specifically, it investigated the distribution and internal consistency of Health Belief Model domains, identified belief-related factors associated with personal screening willingness, and explored factors associated with self-reported screening experience as a secondary outcome.

## 2 Methods

### 2.1 Study design and participants

This cross-sectional study employed convenience sampling to recruit young women from gynecological outpatient clinics in Shenzhen, China. Participants utilized a smartphone QR code to access the Wenjuanxing questionnaire and willingly completed the survey. Due to the absence of data on the individuals exposed to the recruiting information and the QR-code scans, a standard response rate could not be determined. Data acquisition occurred from May 2023 to May 2026. Eligibility criteria included Chinese nationality, providing informed consent, and self-identification as a young woman attending a gynecology outpatient clinic. Because the online questionnaire did not enforce a strict age or education eligibility filter, the final analytic sample included 88 participants (3.8%) who reported below-college education. The sample should therefore be described as a clinic-based sample of predominantly young, college-educated women rather than as representative of all young women in China. Participants with below-college education were retained in the primary available-data analysis and addressed in an education-restricted sensitivity analysis. The analytical dataset lacked directly identifiable variables or functional IP address data. The *Wenjuanxing* platform was configured to limit repeated submissions from the same WeChat account where possible; however, because the exported dataset contained no identifiable IP addresses or device identifiers, IP-based or device-based duplicate detection could not be independently verified, and no formal straight-lining or patterned-response algorithm was applied. The coding for the questionnaire and the mapping of HBM items to domains can be found in Supplementary Table S1; the protocols for data cleaning and handling missing data are detailed in Supplementary Table S2. All accessible questionnaire records containing comprehensive data for the pertinent outcome and HBM items were preserved for the primary analyses of available data. The participant flow and data-cleaning steps are shown in Supplementary Figure S1, and the distributions of age and questionnaire completion time are shown in Supplementary Figure S2.

### 2.2 Measures

#### 2.2.1 Questionnaire development and pilot administration

The questionnaire was created by the study team to evaluate knowledge, attitudes, practices about cervical cancer, beliefs associated with the Health Belief Model, and understanding of HPV vaccine. Items were developed based on prior HBM literature on CCS and HPV prevention, and the item pool was reviewed by the research team for content relevance and clarity. Prior to the official survey, 50 qualified young women participated in a preliminary administration to evaluate item understanding and practical applicability. Pilot evaluations were utilized to assess the phrasing and implementation procedure, and pilot replies were excluded from the ultimate analytical dataset. Item-specific expert content-validity indices were absent for the current report and hence were neither computed nor documented (The HBM domain scores should be regarded as investigator-constructed summary scores).

#### 2.2.2 Demographic and screening-related measures

Demographic variables included age, residence, marital status, education level, chronic disease status, history of cervical disease, family history of cervical disease, and HPV vaccination status. Age was converted to numeric values. Six non-numeric, header-like, or implausible age entries were treated as missing; these records were retained for analyses not requiring age and excluded by complete-case analysis from models including age. Screening-related variables included self-reported screening within the previous three years, number of screening experiences, screening interval, active seeking of screening knowledge, active learning of screening policy, and personal willingness to undergo screening.

The primary outcome was personal willingness to undergo CCS, assessed using the practice-questionnaire item “Would you be willing to undergo CCS?” Responses were coded as willing (“yes,” *n* = 2,046) versus unwilling (“no,” *n* = 271). The use of a single binary item to assess screening willingness is a limitation, as it may not capture the full spectrum of willingness; this is addressed in the Discussion. The separate item asking whether the participant or her family would be willing to undergo screening was not used as the primary outcome. Self-reported screening within the previous three years was treated as an exploratory secondary outcome and was not interpreted as guideline-concordant adherence because sexual history, clinician recommendation, and individual eligibility were not assessed.

#### 2.2.3 Health Belief Model domains

Screening-related health beliefs were assessed using 33 Likert-type items scored from 1 to 5. Items were summarized into seven domains: perceived susceptibility (5 items), perceived severity (5 items), perceived benefits (4 items), perceived barriers (7 items), cues to action (3 items), self-efficacy (3 items), and health motivation (6 items). The item-to-domain mapping is provided in Supplementary Table S1. Domain scores were calculated as the mean of the constituent items. In the present scoring, higher perceived-barrier scores represent stronger perceived barriers. A six-item screening-attitude score was also calculated by averaging the six attitude items. Because some attitude items may be conceptually related to screening willingness, the attitude score was included as a covariate rather than as a primary predictor. Because the seven HBM domains represent conceptually distinct constructs and perceived barriers are directionally different from the other domains, no total HBM composite score was interpreted as a unidimensional measure.

### 2.3 Statistical analysis

Categorical variables were summarized as frequencies and percentages. Continuous or ordinal scale variables were summarized using means and standard deviations together with medians and interquartile ranges as appropriate. Group differences by personal screening willingness were examined using chi-square or Fisher exact tests for categorical variables and Mann–Whitney U tests for scale variables. Internal consistency was assessed using Cronbach’s alpha. All 2,317 records had complete primary-outcome and HBM-item data. Questionnaire completion time was examined as an auxiliary data-quality indicator. The minimum observed completion time was 72 s. Because no prespecified time-based exclusion threshold was available and no completion-time sensitivity analysis was undertaken, questionnaire duration was not used as a primary exclusion criterion. Because the group comparisons across HBM domains were exploratory and involved multiple domains, no formal multiple-comparison correction was applied; *p* values should be interpreted together with effect sizes, and these findings should be regarded as hypothesis-generating.

Multivariable binary logistic regression was used to identify factors associated with personal screening willingness. Candidate predictors included age, residence, marital status, chronic disease status, family history of cervical disease, HPV vaccination status, screening-attitude score, and the seven Health Belief Model domains. HBM domain scores and the screening-attitude score were treated as continuous predictors. The linearity of the logit assumption for continuous predictors was assessed using the Box-Tidwell test; results are reported in Supplementary Table S3. Evidence of nonlinearity was observed for the screening-attitude score and perceived susceptibility (both *p* < 0.05). Because the screening-attitude score was included only as a covariate and perceived susceptibility was not independently associated with the outcome, continuous linear terms were retained in the primary model, and these estimates should be interpreted with this caveat. History of cervical disease was described in the baseline table but excluded from the primary model because only 19 participants reported such a history, creating sparse cells and potentially unstable estimation. The primary model used 2,311 complete cases because age was included. Reference categories were urban residence, unmarried status, no chronic disease, no family history of cervical disease, and no HPV vaccination. VIF, tolerance, and model-fit indices (likelihood-ratio test, AIC, BIC, McFadden R2, Cox and Snell R2, and Nagelkerke R2) for the primary model are reported in Supplementary Table S3. Discrimination was summarized by the area under the receiver-operating-characteristic curve (AUC = 0.777, 95% CI 0.752–0.801). A sensitivity analysis restricted the sample to participants with college/undergraduate education or above. This restriction yielded 2,229 records, of which 2,226 had complete data for the age-adjusted logistic regression model. The education-restricted sensitivity estimates are reported in Table 1. In a further sensitivity analysis that excluded the screening-attitude score, the direction and magnitude of the main associations were unchanged (adjusted OR for perceived barriers 0.453, 95% CI 0.374–0.548; cues to action 1.403, 1.193–1.650; HPV vaccination 1.588, 1.171–2.153; perceived severity 1.253, 1.034–1.517; perceived benefits 1.202, 1.025–1.411). A separate exploratory model for self-reported screening within the previous three years included history of cervical disease. This model should not be interpreted as an assessment of guideline-concordant screening behavior, as screening eligibility was not measured. All associations were interpreted as non-causal because of the cross-sectional design.

**Table 1:** Primary and education-restricted sensitivity models for personal willingness to undergo screening.

Predictor	Primary model aOR (95% CI)	*p* value	College-or-above sensitivity aOR (95% CI)	*p* value
Age	1.042 (0.970–1.119)	0.263	1.010 (0.933–1.094)	0.799
Rural residence	0.857 (0.651–1.127)	0.270	0.843 (0.636–1.117)	0.234
Married/divorced/widowed	2.300 (0.583–9.065)	0.234	5.018 (0.536–47.013)	0.158
Chronic disease	1.123 (0.499–2.524)	0.780	1.224 (0.519–2.889)	0.644
Family history of cervical disease	0.605 (0.286–1.278)	0.188	0.655 (0.300–1.432)	0.289
HPV vaccinated	1.576 (1.161–2.137)	0.003	1.572 (1.152–2.147)	0.004
Screening attitude score	1.097 (0.950–1.265)	0.207	1.091 (0.943–1.262)	0.244
Perceived susceptibility	0.889 (0.741–1.068)	0.210	0.864 (0.716–1.043)	0.128
Perceived severity	1.257 (1.038–1.523)	0.019	1.255 (1.030–1.528)	0.024
Perceived benefits	1.194 (1.017–1.402)	0.030	1.201 (1.019–1.415)	0.029
Perceived barriers	0.454 (0.375–0.550)	**<**0.001	0.460 (0.378–0.559)	**<**0.001
Cues to action	1.399 (1.189–1.646)	**<**0.001	1.392 (1.178–1.644)	**<**0.001
Self-efficacy	1.149 (0.949–1.391)	0.154	1.154 (0.948–1.405)	0.154
Health motivation	1.100 (0.908–1.331)	0.330	1.100 (0.904–1.337)	0.342

The primary model included 2,311 complete cases; the education-restricted sensitivity model included 2,226 complete cases from 2,229 participants with college/undergraduate education or above. Odds ratios represent associations, not causal effects. Key estimates were similar across models.

## 3 Results

### 3.1 Participant characteristics

A total of 2,317 questionnaire records were available for analysis. All records contained complete data for the primary outcome and the 33 HBM items. Six records had invalid or non-numeric age values; therefore, age-specific analyses used 2,311 valid observations, whereas analyses not requiring age retained all 2,317 records. Among valid age observations, the median age was 20 years (IQR 19–21), and 2,238 (96.8%) were aged 18–24 years. Most participants were unmarried (2,250; 97.1%). College/undergraduate education was reported by 2,212 participants (95.5%). Including participants with postgraduate education, 2,229 participants (96.2%) had college education or above, whereas 88 participants (3.8%) had below-college education. Rural and urban residence were nearly balanced. Participant characteristics and bivariate comparisons by screening willingness are presented in Table 2; data-cleaning details are provided in Supplementary Table S2 and the participant/data-cleaning flow is shown in Supplementary Figure S1.

**Table 2:** Participant characteristics by personal willingness to undergo CCS.

Characteristic	Overall (*N* = 2,317)	Unwilling (*n* = 271)	Willing (*n* = 2046)	*p* value
Age, median (IQR)	20 (19–21)	20 (19–21)	20 (19–21)	0.369
Age group				0.337
≤17 years	5 (0.2)	0 (0.0)	5 (0.2)	
18–24 years	2,238 (96.8)	266 (98.2)	1972 (96.4)	
25–34 years	32 (1.4)	3 (1.1)	29 (1.4)	
35–44 years	31 (1.3)	0 (0.0)	31 (1.5)	
45–54 years	3 (0.1)	0 (0.0)	3 (0.1)	
≥55 years	2 (0.1)	0 (0.0)	2 (0.1)	
Residence				0.098
Urban	1,131 (48.8)	119 (43.9)	1,012 (49.5)	
Rural	1,186 (51.2)	152 (56.1)	1,034 (50.5)	
Marital status				0.204
Unmarried	2,250 (97.1)	267 (98.5)	1983 (96.9)	
Married	57 (2.5)	2 (0.7)	55 (2.7)	
Divorced	6 (0.3)	1 (0.4)	5 (0.2)	
Widowed	4 (0.2)	1 (0.4)	3 (0.1)	
Education				0.065
Primary school or below	8 (0.3)	3 (1.1)	5 (0.2)	
Junior high school	19 (0.8)	0 (0.0)	19 (0.9)	
High school/technical secondary school	61 (2.6)	9 (3.3)	52 (2.5)	
College/undergraduate	2,212 (95.5)	256 (94.5)	1956 (95.6)	
Postgraduate or above	17 (0.7)	3 (1.1)	14 (0.7)	
Chronic disease				1.0
No	2,243 (96.8)	262 (96.7)	1981 (96.8)	
Yes	74 (3.2)	9 (3.3)	65 (3.2)	
Family history of cervical disease				0.41
No	2,246 (96.9)	260 (95.9)	1986 (97.1)	
Yes	71 (3.1)	11 (4.1)	60 (2.9)	
HPV vaccinated				0.166
No	1,560 (67.3)	193 (71.2)	1,367 (66.8)	
Yes	757 (32.7)	78 (28.8)	679 (33.2)	

Age analyses used 2,311 valid numeric age observations; six age entries were treated as missing or invalid. Age-group percentages are based on valid age observations. *p* values were obtained from the Mann–Whitney U test for age and chi-square test or Fisher exact test for categorical variables, as appropriate. *p* values refer to overall comparisons for each characteristic. Percentages in the unwilling and willing columns are calculated within each willingness group.

### 3.2 Cervical cancer screening willingness and experience

Past CCS experience was uncommon, with 219 participants (9.5%) reporting screening within the previous three years. Personal willingness to undergo screening was reported by 2,046 participants (88.3%). Active information-seeking was also common: 63.2% had actively sought screening-related knowledge and 62.1% had actively learned about screening-related policies. Table 3 summarizes screening experience, information-seeking, and willingness; Figure 1 presents these two descriptive indicators. Because individual screening eligibility was not assessed and the two items concern different constructs (previous screening experience and general future willingness), their difference should not be interpreted as a willingness–behavior gap or as poor adherence.

**Table 3:** CCS experience, information-seeking, and personal willingness.

Screening willingness indicator	Category	*n*	%
Past CCS within 3 years	No	2098	90.5
	Yes	219	9.5
Number of lifetime screenings	Never	2094	90.4
	Once	129	5.6
	Twice	51	2.2
	Three or more times	43	1.9
Self-reported screening interval	Never	2099	90.6
	Every 3 years	156	6.7
	Less often than every 3 years	62	2.7
Actively sought screening knowledge	No	853	36.8
	Yes	1,464	63.2
Actively learned screening policy	No	877	37.9
	Yes	1,440	62.1
Future screening willingness	Unwilling	271	11.7
	Willing	2046	88.3

Percentages use the full analytic sample (*N* = 2,317). Screening experience is self-reported and exploratory because sexual history and individual guideline-based eligibility were not assessed.

**Figure 1:**
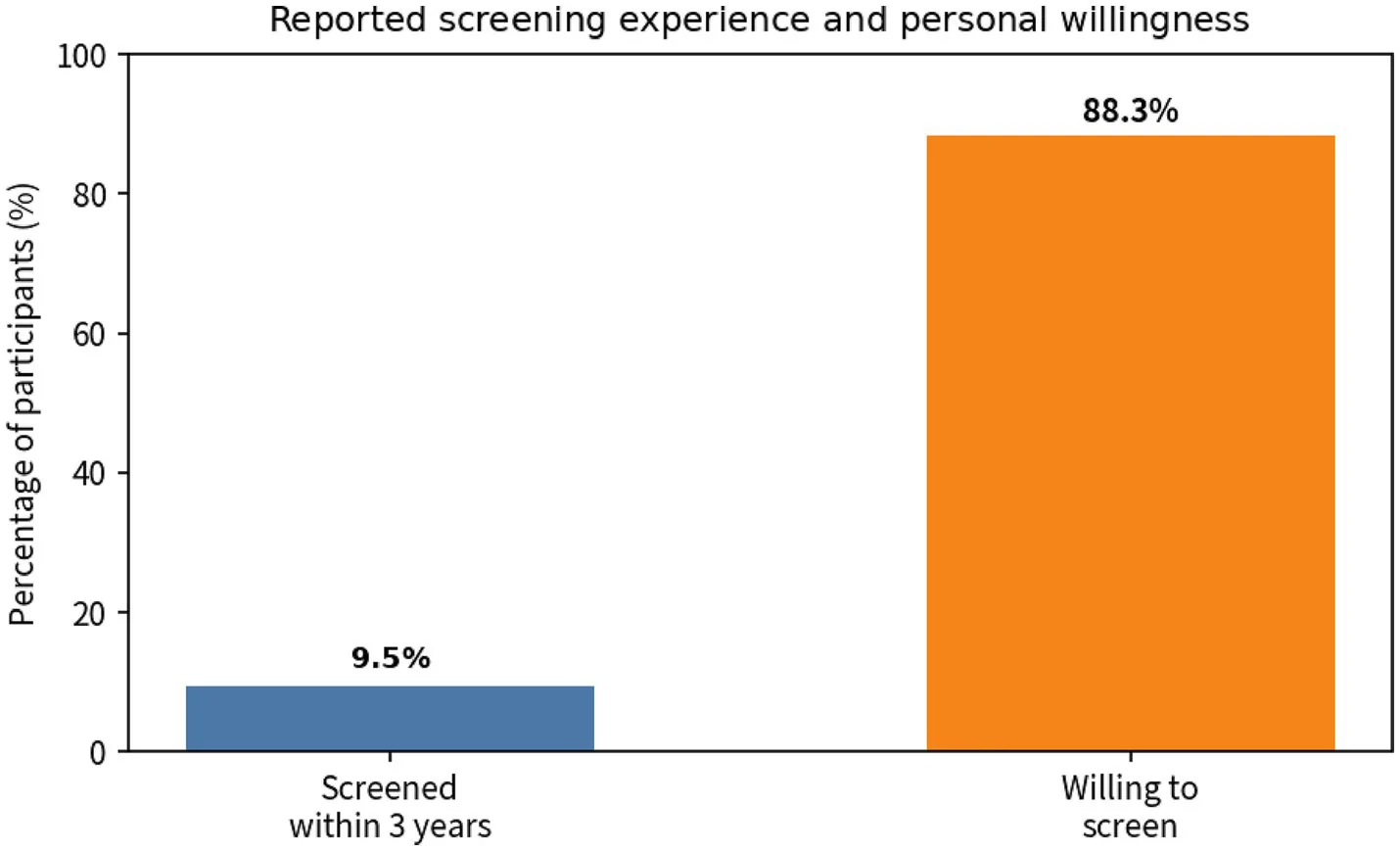
Reported screening experience and personal willingness to undergo cervical cancer screening. Percentages are based on *n* = 2,317. The two indicators represent different self-reported constructs and should not be interpreted as a longitudinal transition, an intention–behavior gap, or a willingness-behavior gap.

### 3.3 Distribution and internal consistency of Health Belief Model domains

The Health Belief Model scale showed strong internal consistency. Cronbach alpha values ranged from 0.890 to 0.965 across the seven domains. No total HBM composite score was interpreted as a unidimensional measure. The highest domain score was observed for health motivation, followed by cues to action, perceived benefits, and self-efficacy. Perceived susceptibility and perceived barriers had comparatively lower mean scores. Table 4 presents the domain distributions and reliability estimates; item-level reliability diagnostics are reported in Supplementary Table S4. Sampling adequacy and exploratory construct-validity results of the HBM scale are provided in Supplementary Table S5.

**Table 4:** Distribution and internal consistency of Health Belief Model domains.

Scale/domain	Items	Mean (SD)	Median (IQR)	Cronbach alpha
Perceived susceptibility	5	1.91 (1.15)	1.40 (1.00–2.60)	0.952
Perceived severity	5	2.62 (1.29)	2.80 (1.00–3.40)	0.915
Perceived benefits	4	3.79 (1.26)	4.00 (3.00–5.00)	0.890
Perceived barriers	7	2.26 (1.06)	2.14 (1.43–2.86)	0.903
Cues to action	3	3.93 (1.25)	4.00 (3.00–5.00)	0.945
Self-efficacy	3	3.73 (1.13)	4.00 (3.00–5.00)	0.898
Health motivation	6	4.28 (1.01)	4.83 (4.00–5.00)	0.965

Domain scores range from 1 to 5. Higher scores indicate stronger endorsement of the named construct; for perceived barriers, higher scores indicate stronger perceived barriers. The complete scoring protocol is reported in Supplementary Table S1. Because the seven domains represent conceptually distinct constructs and perceived barriers are directionally different from the other domains, no total HBM composite score is reported or interpreted as a unidimensional measure.

Principal component analysis (PCA) with Varimax rotation was used to examine the dimensionality of the 33 HBM items. Seven components were requested *a priori* based on the theoretical HBM framework. The data showed excellent sampling adequacy (KMO = 0.942) and a significant Bartlett’s test of sphericity (χ^2^ = 80,628.149, df = 528, *p* < 0.001). The seven requested components explained 80.337% of the total variance; however, the seventh component had an eigenvalue of 0.889, below the conventional threshold of 1.00. Several items showed cross-loadings across components (q6, q11, q18, q21, q24, q25, q26, and q27). Therefore, these findings provide only preliminary evidence regarding the compatibility of the item structure with the theoretical HBM framework and do not constitute full validation of a seven-domain measurement model.

### 3.4 Health belief domains by willingness to undergo future screening

Participants willing to undergo personal CCS had higher median scores for perceived benefits, cues to action, self-efficacy, health motivation, and screening attitudes, and lower perceived-barrier scores than unwilling participants. Perceived susceptibility also differed between groups, but the median score was lower among willing participants and the effect was small (r = 0.066). Perceived severity did not differ between groups. Overall, the statistically significant group differences were small in magnitude. Effect sizes were small to moderate, indicating that statistical significance should not be equated with large practical effects. The complete group comparisons are reported in Table 5, and the HBM domain profile is displayed in Figure 2.

**Table 5:** Health Belief Model domains by personal willingness to undergo screening.

Domain	Unwilling median (IQR)	Willing median (IQR)	U statistic	*P* value	Effect size r
Screening attitude score	4.50 (3.50–5.00)	5.00 (4.33–5.00)	218493.5	<0.001	0.118
Perceived susceptibility	1.80 (1.00–3.00)	1.40 (1.00–2.60)	309929.0	<0.001	0.066
Perceived severity	2.80 (1.00–3.60)	2.80 (1.00–3.40)	275835.5	0.892	0.003
Perceived benefits	3.25 (3.00–5.00)	4.00 (3.00–5.00)	216757.5	<0.001	0.121
Perceived barriers	2.86 (1.64–3.29)	2.14 (1.43–2.71)	345552.0	<0.001	0.137
Cues to action	3.00 (2.50–5.00)	4.33 (3.33–5.00)	195507.0	<0.001	0.164
Self-efficacy	3.00 (2.67–4.00)	4.00 (3.00–5.00)	210350.0	<0.001	0.134
Health motivation	4.00 (3.00–5.00)	4.83 (4.00–5.00)	193789.0	<0.001	0.168

Values are median (IQR). *p* values are from two-sided Mann–Whitney U tests. Because these comparisons were exploratory and involved multiple HBM domains, no formal multiple-comparison correction was applied; *p* values should be interpreted together with effect sizes, and these comparisons should be regarded as hypothesis-generating. Effect sizes are reported as r. The total HBM scale is not reported because the seven domains represent conceptually distinct constructs and perceived barriers are directionally different from the other domains.

**Figure 2:**
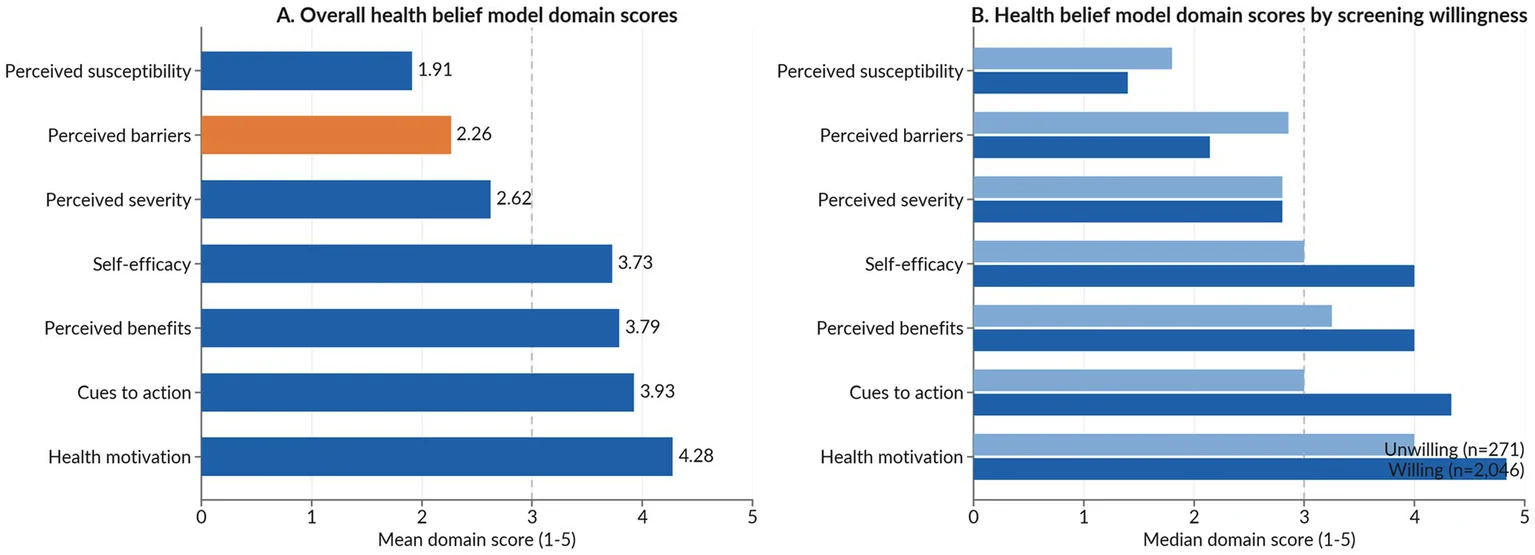
Health Belief Model domain profile by personal willingness to undergo screening. **(A)** Overall mean domain scores; **(B)** domain scores by self-reported screening willingness. Bars represent means in panel **(A)** and medians in panel **(B)**. Higher perceived-barrier scores indicate stronger perceived barriers. Colors are selected from a color-vision-deficiency-friendly palette.

### 3.5 Multivariable factors associated with future screening willingness

In the multivariable logistic regression model (*n* = 2,311), perceived barriers were inversely associated with personal screening willingness. Cues to action, perceived severity, perceived benefits, and HPV vaccination status were positively associated with willingness after adjustment. Screening-attitude score, self-efficacy, health motivation, residence, marital status, chronic disease, and family history of cervical disease were not independently associated with willingness. The adjusted estimates are reported in Table 6 and visualized in Figure 3. Model-fit indices and collinearity diagnostics are reported in Supplementary Table S3. The education-restricted sensitivity estimates are presented in Table 1. The education-restricted sensitivity model, which restricted the sample to participants with college/undergraduate education or above (*n* = 2,226 for the complete-case regression), produced estimates similar to those of the primary model for HPV vaccination status, perceived severity, perceived benefits, perceived barriers, and cues to action, supporting the robustness of the main findings to the education-target discrepancy; these estimates are presented in Table 1. Figure 4 presents the descriptive distribution of screening willingness by HPV vaccination status; this comparison is not causal. In the Box–Tidwell assessment, no statistically significant departure from linearity was detected for perceived barriers, cues to action, perceived severity, or perceived benefits (all *p* > 0.15), whereas evidence of nonlinearity was observed for the screening-attitude covariate and perceived susceptibility (both *p* < 0.05; Supplementary Table S3). Because the attitude score was included only as a covariate and perceived susceptibility was not independently associated with the outcome, the primary model retained continuous linear terms, and these estimates should be interpreted with this caveat.

**Table 6:** Multivariable logistic regression for personal willingness to undergo screening.

Predictor	Adjusted OR (95% CI)	*p* value
Age	1.042 (0.970–1.119)	0.263
Rural residence	0.857 (0.651–1.127)	0.270
Married/divorced/widowed	2.300 (0.583–9.065)	0.234
Chronic disease	1.123 (0.499–2.524)	0.780
Family history of cervical disease	0.605 (0.286–1.278)	0.188
HPV vaccinated	1.576 (1.161–2.137)	0.003
Screening attitude score	1.097 (0.950–1.265)	0.207
Perceived susceptibility	0.889 (0.741–1.068)	0.210
Perceived severity	1.257 (1.038–1.523)	0.019
Perceived benefits	1.194 (1.017–1.402)	0.030
Perceived barriers	0.454 (0.375–0.550)	<0.001
Cues to action	1.399 (1.189–1.646)	<0.001
Self-efficacy	1.149 (0.949–1.391)	0.154
Health motivation	1.100 (0.908–1.331)	0.330

The model included 2,311 complete cases. Reference groups were urban residence, unmarried status, no chronic disease, no family history of cervical disease, and no HPV vaccination. Odds ratios represent associations, not causal effects. Model fit: Nagelkerke R2 = 0.178; area under the receiver-operating-characteristic curve (AUC) = 0.777 (95% CI 0.752–0.801). The Hosmer-Lemeshow test was not performed. VIF and tolerance are reported in Supplementary Table S3.

**Figure 3:**
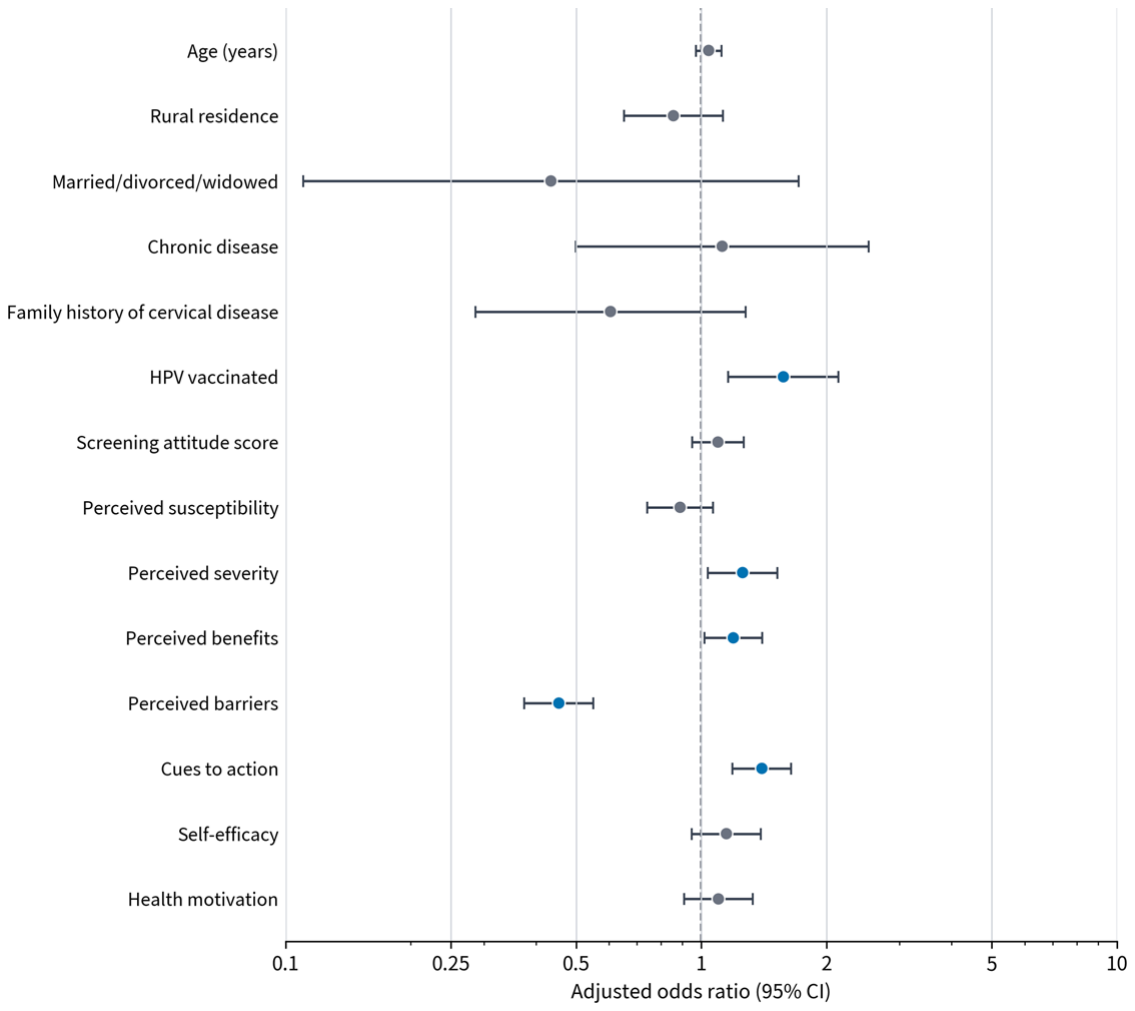
Adjusted odds ratios for factors associated with personal willingness to undergo screening. Points and intervals represent adjusted odds ratios and 95% confidence intervals. The log scale is used for the odds-ratio axis. Associations are non-causal. The wide confidence interval for the married/divorced/widowed category reflects the small number of participants in this group and should be interpreted with caution.

**Figure 4:**
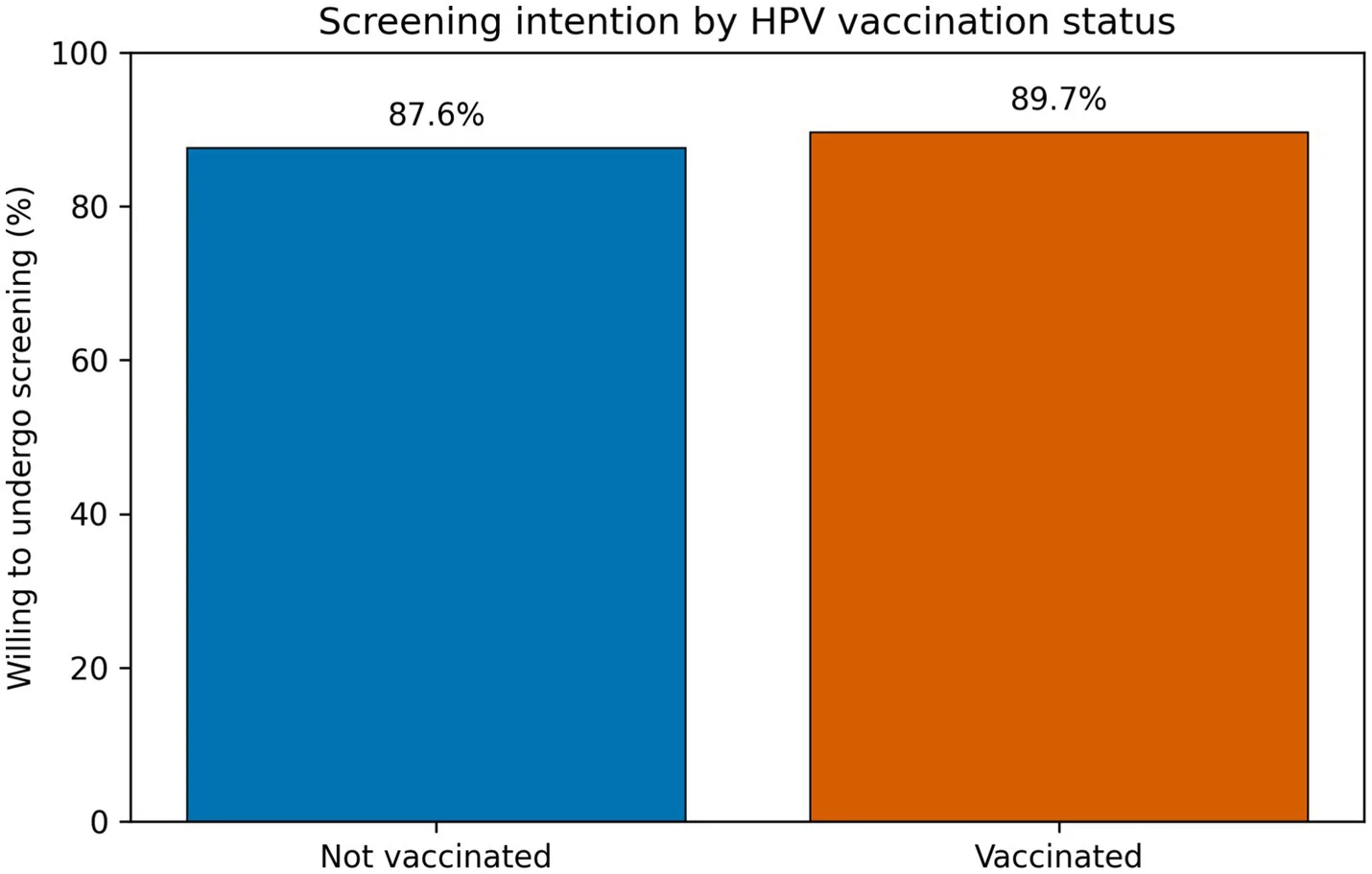
Personal willingness to undergo screening by HPV vaccination status. Percentages are calculated within HPV vaccination-status groups using the available cross-tabulation. This descriptive comparison does not establish a causal effect of vaccination.

### 3.6 Exploratory model for past screening experience

Past screening experience was analyzed as a secondary exploratory outcome and should not be interpreted as an indicator of guideline adherence. Older age, HPV vaccination, prior cervical disease history, perceived susceptibility, and self-efficacy were positively associated with reporting screening within the previous three years. Health motivation was inversely associated with past screening after adjustment. These exploratory estimates are reported in Table 7 and should be interpreted with the model diagnostics and sensitivity analyses in Supplementary Table S3. Because screening eligibility was not measured, this model does not represent guideline-concordant screening behavior.

**Table 7:** Exploratory multivariable logistic regression for self-reported screening experience.

Predictor	Adjusted OR (95% CI)	*p* value
Age	1.104 (1.051–1.158)	<0.001
Rural residence	1.094 (0.806–1.483)	0.564
Married/divorced/widowed	1.723 (0.703–4.221)	0.234
Chronic disease	1.126 (0.480–2.642)	0.785
History of cervical disease	7.119 (1.848–27.431)	0.004
Family history of cervical disease	0.760 (0.316–1.832)	0.542
HPV vaccinated	3.568 (2.610–4.876)	<0.001
Screening attitude score	0.906 (0.767–1.071)	0.248
Perceived susceptibility	1.495 (1.215–1.841)	<0.001
Perceived severity	0.897 (0.728–1.107)	0.312
Perceived benefits	0.924 (0.782–1.092)	0.354
Perceived barriers	0.935 (0.770–1.135)	0.494
Cues to action	0.863 (0.728–1.023)	0.090
Self-efficacy	1.526 (1.197–1.946)	<0.001
Health motivation	0.738 (0.570–0.956)	0.022

This exploratory model concerns self-reported screening experience and must not be interpreted as a model of guideline-concordant adherence. Estimates involving sparse clinical-history categories require caution.

### 3.7 Summary of results

The results show that the sample represented a predominantly college-educated young female population with limited prior CCS experience and substantial future screening willingness. The low prevalence of past screening should be interpreted in light of the young age structure and unknown screening eligibility rather than as poor adherence, and the difference between past screening and future willingness should not be interpreted as a willingness-behavior gap. Health belief factors, particularly perceived barriers and cues to action, were central to self-reported screening willingness (18). These results identify perceived barriers and cues to action as factors associated with self-reported screening willingness. They suggest potential targets for guideline-based prevention communication, but because the design is cross-sectional, they do not demonstrate that modifying these factors will increase willingness or screening uptake (19). This study should be interpreted as an assessment of reported screening willingness and modifiable prevention beliefs in a clinic-recruited sample, rather than as an estimate of screening coverage or behavior in the broader population of young women in China. The distinction matters because screening willingness is upstream of behavior, and because a young participant may not yet be eligible for screening under applicable guidelines. The findings nevertheless have practical value: a clinical encounter can be used to establish accurate prevention knowledge, clarify the difference between HPV vaccination and CCS, and prepare patients to use screening at the appropriate time (20, 21).

## 4 Discussion

### 4.1 Principal findings and interpretation

This predominantly college-educated young sample reported both limited prior screening experience and substantial personal willingness to undergo screening in the future. Because individual screening eligibility was not measured, the 9.5% prevalence of screening within the previous three years cannot be interpreted as poor adherence, nor can the difference between the two indicators be interpreted as a willingness-behavior gap. Instead, the findings characterize a prevention stage in which many participants expressed willingness to screen in the future while their prior clinical exposure to screening remained limited. This distinction is important because willingness is an upstream behavioral construct, not evidence of guideline-concordant screening behavior (22).

### 4.2 Health Belief Model correlates of screening willingness

Perceived barriers showed the strongest inverse association with personal screening willingness, whereas cues to action, perceived severity, perceived benefits, and HPV vaccination status were positively associated with willingness after adjustment (23). These associations are consistent with a prevention framework in which perceived inconvenience, embarrassment, pain, cost, distance, and fatalistic beliefs may discourage future participation even when general attitudes are favorable (1). The findings should be interpreted as cross-sectional associations, not causal effects. The small-to-moderate magnitude of the unadjusted group differences also indicates that statistical significance should not be equated with large practical effects.

### 4.3 Implications for clinic-linked health education

The association with cues to action suggests that outpatient encounters may be a useful setting for prevention communication, but this remains a hypothesis (24). A potentially useful cue would need to be specific, trusted, and connected to a feasible next step; in gynecology outpatient services, this might include clinician-delivered explanations of guideline-based screening timing, privacy and procedure expectations, and navigation to appropriate services (10). Digital messages or QR-code resources may reinforce consultation, but should direct participants to authoritative sources rather than unverified online content (25, 26). These possibilities should be tested prospectively because the present data do not establish that they will increase screening uptake.

### 4.4 Integrating HPV vaccination and screening messages

HPV vaccination status was positively associated with screening willingness, which may reflect greater prevention awareness, more frequent contact with health services, or both. Vaccination and screening should nevertheless be communicated as complementary rather than interchangeable strategies (27). Health education could explain that HPV vaccination does not eliminate the need for future guideline-based screening, while avoiding messages that imply that all young women require screening immediately (28). Risk communication could also balance the seriousness of CC against reassurance about prevention and the availability of appropriate care (29). These implications are tentative because the present study is cross-sectional.

### 4.5 Interpretation of the exploratory screening-experience model

Older age, HPV vaccination, prior cervical disease, perceived susceptibility, and self-efficacy were associated with reported previous screening. These findings may indicate greater perceived personal relevance or greater contact with prevention services (30). However, this model does not measure guideline-concordant screening because sexual history, clinician recommendation, indication for testing, and diagnostic follow-up were unavailable. The wide confidence interval for cervical disease history also reflects a sparse subgroup. Future studies should distinguish organized screening, opportunistic screening, diagnostic examination, and follow-up of abnormal results (31).

### 4.6 Measurement considerations and future research

The high internal-consistency coefficients support the reliability of the item sets in this sample but do not independently establish construct validity or confirm the proposed seven-domain structure. The principal component analysis results indicate excellent sampling adequacy but require cautious interpretation because the requested seventh component had an eigenvalue below 1 and some cross-loadings were observed (32–34). The proposed seven-domain structure should therefore be regarded as preliminary and confirmed by confirmatory factor analysis in an independent sample, reporting the estimation method, factor loadings, model-fit indices, and any prespecified model modifications (35). Prospective studies should test whether barrier-focused counseling and clinician-linked cues improve guideline-appropriate prevention behavior rather than willingness alone (36). Finally, future research may benefit from broader, multilevel models of screening uptake. Traditional individual-level behavioral models such as the HBM are useful for organizing perceptions and beliefs, but they may not fully capture the social, structural, access-related, and health-system conditions that enable or constrain actual screening (37, 38). Integrated or consolidated screening-uptake models that incorporate individual perceptions together with provider, community, organizational, and policy-level determinants may be particularly valuable when moving from the study of screening willingness to understanding actual screening uptake and when designing interventions that need to address barriers that cannot be modified through education alone (39–43).

### 4.7 Limitations

Several limitations should be noted. First, the cross-sectional design does not allow for causality inference; all associations should be interpreted as correlational. Second, volunteer bias might have been introduced during convenience recruitment in gynecology outpatient clinics, as those who decided to participate might have had a greater interest in CC prevention or health-related topics than those who did not participate. Clinic-based recruitment may bias the sample toward young women with recent healthcare contact; findings may thus be less generalizable to community-based populations of the same age with no recent healthcare contact. This selection process may have resulted in a higher observed screening willingness than would be expected in a broader community-based population. Third, although recruitment targeted a higher-education population, 88 participants (3.8%) reported below-college education; this subgroup was retained in the primary analysis and addressed with an education-restricted sensitivity analysis. Fourth, the primary outcome was assessed with a single binary item (“Would you be willing to undergo CCS?”) that does not capture the intensity, timing, or contextual dimensions of screening willingness and could be subject to social desirability bias. Fifth, the survey did not collect information on sexual activity, age at first sexual intercourse, clinician recommendation, or other information needed to determine individual eligibility for CCS. Thus, previous screening experience could not be interpreted as adherence to guidelines. Sixth, the screening history and stated willingness were self-reported, which may be influenced by recall or social desirability bias. Seventh, although a pilot administration of 50 participants was undertaken to examine questionnaire understanding and feasibility, detailed item-level content-validity records were not available for this report. The measurement structure should be further evaluated in the future with independent samples and a documented validation protocol. Eighth, the low number of participants with prior cervical disease (*n* = 19) limited the stability of some regression estimates. Ninth, data collection occurred over a three-year period; recruitment timing was not recorded by calendar year and was not examined as a source of heterogeneity, although changes in HPV vaccination uptake, public awareness, and screening policy during this period could have influenced responses. Tenth, duplicate, patterned, and inattentive responses could not be formally detected because the survey platform did not provide identifiable IP or device data and no straight-lining algorithm was applied. Eleventh, educational diversity was limited, as most participants held a college or undergraduate degree. Finally, the study discusses implications for prevention communication, but does not directly measure interpersonal communication and diffusion effects. In addition, the Box-Tidwell test indicated evidence of nonlinearity for the screening-attitude covariate and perceived susceptibility; because the attitude score was included only as a covariate and perceived susceptibility was not independently associated with the outcome, continuous linear terms were retained, and this should be considered a limitation. Two of the six screening-attitude items also concerned behavioral willingness and therefore overlapped conceptually with the primary outcome; although a sensitivity analysis excluding the attitude score yielded similar estimates, some residual overlap cannot be excluded.

## 5 Conclusion

In this predominantly young and college-educated clinic-based sample in Shenzhen, China, personal willingness to undergo CCS was commonly reported, whereas prior screening experience was limited. Perceived barriers and cues to action were associated with screening willingness, but causal effects cannot be inferred, and the present study cannot determine whether modifying these factors would increase willingness or screening uptake. Future studies should evaluate guideline-based prevention communication, barrier reduction, and appropriate screening timing in more representative community samples. These findings identify potential targets for clinic-linked health education, but prospective or intervention studies are needed to establish whether addressing perceived barriers and leveraging cues to action can promote timely, guideline-concordant screening. The seven-domain Health Belief Model structure is based on investigator-constructed summary scores and exploratory dimensionality analysis and therefore requires confirmation in independent samples before it can be regarded as validated.

## Data Availability

The raw data supporting the conclusions of this article will be made available by the authors, without undue reservation.
