# Supplementary material for: Cervical cancer screening willingness and associated health beliefs among predominantly college-educated young women attending gynecology clinics in Shenzhen, China
Source: Front Public Health. 2026 Oct 12;14:1969898. doi: 10.3389/fpubh.2026.1969898 (PMC13696420; doi:10.3389/fpubh.2026.1969898)
Supplement: Supplementary file 1 [file Data_Sheet_1.docx]

**Supplementary Material**

**Supplementary Table S1. Questionnaire coding and Health Belief Model domain mapping**

**Panel A. Questionnaire items and Health Belief Model domain mapping**

| **Item** | **Questionnaire item** | **HBM domain** | **Response options** | **Reverse-scored** |
| --- | --- | --- | --- | --- |
| Q1 | I feel that I will develop cervical cancer in the future. | Perceived susceptibility | 1 = strongly disagree; 5 = strongly agree | No |
| Q2 | I have a high chance of developing cervical cancer. | Perceived susceptibility | 1 = strongly disagree; 5 = strongly agree | No |
| Q3 | I cannot avoid developing cervical cancer. | Perceived susceptibility | 1 = strongly disagree; 5 = strongly agree | No |
| Q4 | I worry about developing cervical cancer because a family member has had cervical cancer. | Perceived susceptibility | 1 = strongly disagree; 5 = strongly agree | No |
| Q5 | I am more likely than the general population to develop cervical cancer. | Perceived susceptibility | 1 = strongly disagree; 5 = strongly agree | No |
| Q6 | I feel anxious when I think about how serious cervical cancer is. | Perceived severity | 1 = strongly disagree; 5 = strongly agree | No |
| Q7 | If I had cervical cancer, treatment costs would create financial pressure for my family. | Perceived severity | 1 = strongly disagree; 5 = strongly agree | No |
| Q8 | Cervical cancer would threaten my relationship with my boyfriend, husband, or sexual partner. | Perceived severity | 1 = strongly disagree; 5 = strongly agree | No |
| Q9 | If I had cervical cancer, my whole life would change. | Perceived severity | 1 = strongly disagree; 5 = strongly agree | No |
| Q10 | If I had cervical cancer, I would not live for more than 5 years. | Perceived severity | 1 = strongly disagree; 5 = strongly agree | No |
| Q11 | Cervical cancer screening helps reduce the risk of developing cervical cancer. | Perceived benefits | 1 = strongly disagree; 5 = strongly agree | No |
| Q12 | Cervical cancer screening helps reduce the risk of death from cervical cancer. | Perceived benefits | 1 = strongly disagree; 5 = strongly agree | No |
| Q13 | Cervical cancer screening can improve my health. | Perceived benefits | 1 = strongly disagree; 5 = strongly agree | No |
| Q14 | Cervical cancer screening would reduce my anxiety about cervical cancer. | Perceived benefits | 1 = strongly disagree; 5 = strongly agree | No |
| Q15 | Cervical cancer screening would make me feel embarrassed because it requires exposing the perineum. | Perceived barriers | 1 = strongly disagree; 5 = strongly agree | No |
| Q16 | Cervical cancer screening takes too much time. | Perceived barriers | 1 = strongly disagree; 5 = strongly agree | No |
| Q17 | Cervical cancer screening is too painful. | Perceived barriers | 1 = strongly disagree; 5 = strongly agree | No |
| Q18 | I am too old to need cervical cancer screening. | Perceived barriers | 1 = strongly disagree; 5 = strongly agree | No |
| Q19 | The hospital providing cervical cancer screening is far from where I live. | Perceived barriers | 1 = strongly disagree; 5 = strongly agree | No |
| Q20 | Cervical cancer screening is costly; I would not undergo it unless it were free. | Perceived barriers | 1 = strongly disagree; 5 = strongly agree | No |
| Q21 | I believe cervical cancer is predetermined; screening would not change this. | Perceived barriers | 1 = strongly disagree; 5 = strongly agree | No |
| Q22 | I would undergo cervical cancer screening if I received social support. | Cues to action | 1 = strongly disagree; 5 = strongly agree | No |
| Q23 | I would undergo cervical cancer screening if I had sufficient information support, such as from the internet, health lectures, radio, or television. | Cues to action | 1 = strongly disagree; 5 = strongly agree | No |
| Q24 | I would undergo cervical cancer screening if I were aware of cervical cancer prevention. | Cues to action | 1 = strongly disagree; 5 = strongly agree | No |
| Q25 | I believe that I can undergo regular cervical cancer screening. | Self-efficacy | 1 = strongly disagree; 5 = strongly agree | No |
| Q26 | I can recognize changes in my body that are normal or abnormal. | Self-efficacy | 1 = strongly disagree; 5 = strongly agree | No |
| Q27 | I know how cervical cancer can be detected through regular screening. | Self-efficacy | 1 = strongly disagree; 5 = strongly agree | No |
| Q28 | I want to identify health risks as early as possible. | Health motivation | 1 = strongly disagree; 5 = strongly agree | No |
| Q29 | Maintaining good health is very important to me. | Health motivation | 1 = strongly disagree; 5 = strongly agree | No |
| Q30 | To maintain my health, I seek and consult information about cervical cancer. | Health motivation | 1 = strongly disagree; 5 = strongly agree | No |
| Q31 | I think health-promoting activities are very important. | Health motivation | 1 = strongly disagree; 5 = strongly agree | No |
| Q32 | For my health, I eat a balanced diet. | Health motivation | 1 = strongly disagree; 5 = strongly agree | No |
| Q33 | For my health, I exercise at least three times per week. | Health motivation | 1 = strongly disagree; 5 = strongly agree | No |

*Note: All items were scored 1 = strongly disagree, 2 = disagree, 3 = uncertain, 4 = agree, and 5 = strongly agree. No items were reverse-scored; higher scores denote stronger endorsement of the named construct (for perceived barriers, stronger perceived barriers). All 33 Health Belief Model items were complete for all 2,317 records, so every domain score was computed from its complete item set. Because the seven HBM domains represent conceptually distinct constructs and perceived barriers are directionally different from the other domains, no total HBM composite score was interpreted as a unidimensional measure.*

**Panel B. Scoring protocol**

| **Score** | **Composition** | **Scale** |
| --- | --- | --- |
| Perceived susceptibility | Mean of items q1–q5 | 1–5 |
| Perceived severity | Mean of items q6–q10 | 1–5 |
| Perceived benefits | Mean of items q11–q14 | 1–5 |
| Perceived barriers | Mean of items q15–q21 | 1–5 |
| Cues to action | Mean of items q22–q24 | 1–5 |
| Self-efficacy | Mean of items q25–q27 | 1–5 |
| Health motivation | Mean of items q28–q33 | 1–5 |
| Screening-attitude score | Mean of the 6 screening-attitude items | 1–5 |

*Note: No total HBM composite score is reported or interpreted as a unidimensional measure, because the seven domains represent conceptually distinct constructs and perceived barriers are directionally different from the other domains.*

**Panel C. Screening-attitude items**

The six screening-attitude items were: (1) various screening methods can detect cervical cancer early; (2) screening helps prevent cervical cancer; (3) cervical cancer screening is not harmful to health; (4) I would undergo screening if cervical cancer screening were free; (5) HPV vaccination can prevent cervical cancer; and (6) I would get vaccinated if the HPV vaccine were free. Each item was scored from 1 to 5, and the screening-attitude score was the mean of the six items. Because items (4) and (6) concern behavioral willingness, the attitude score was included as a covariate rather than as a primary predictor, and a sensitivity analysis excluding the score was performed (main text, Section 2.3).

**Supplementary Table S2. Data cleaning, missingness, and excluded records**

**Panel A. Data-cleaning summary**

| **Rule** | **n** | **%** |
| --- | --- | --- |
| Raw records | 2317 | 100.000% |
| Invalid/non-numeric/implausible age entries | 6 | 0.259% |
| Valid numeric age observations | 2311 | 99.741% |
| Past screening within previous 3 years: yes | 219 | 9.450% |
| Personal screening willingness: willing | 2046 | 88.300% |
| HPV vaccinated: yes | 757 | 32.670% |
| Below-college education (retained in primary available-data sample) | 88 | 3.800% |

**Panel B. Variables with missing or special values**

| **Column(s)** | **Source variable(s)** | **Missing/special values, n** | **Missing/special values, %** | **Observed levels** |
| --- | --- | --- | --- | --- |
| 2 | Completion time | 0 | 0.0000 | 777 |
| 3 | Total questionnaire score (platform-computed) | 0 | 0.0000 | 124 |
| 4 | Survey introductory text | 2,252 | 97.1900 | 42 |
| 5 | General information section header | 2,289 | 98.7900 | 17 |
| 6 | Age | 0 | 0.0000 | 35 |
| 7-15 | Demographic and screening-history items | 0 | 0.0000 | 2-5 |
| 16 | Cervical cancer knowledge section header | 2,292 | 98.9200 | 11 |
| 17-31 | Cervical cancer knowledge items (15 items) | 0 | 0.0000 | 3-5 |
| 32 | Cervical cancer screening attitude section header | 2,296 | 99.0900 | 15 |
| 33-38 | Screening-attitude items (6 items) | 0 | 0.0000 | 5 |
| 39 | Cervical cancer screening practice section header | 2,303 | 99.4000 | 10 |
| 40-45 | Screening-practice items (6 items) | 0 | 0.0000 | 2-4 |
| 46 | Chinese Health Belief Model scale section header | 2,308 | 99.6100 | 9 |
| 47-79 | Health Belief Model items (33 items) | 0 | 0.0000 | 5 |
| 80 | HPV vaccine awareness questionnaire section header | 2,303 | 99.4000 | 9 |
| 81-105 | HPV vaccine awareness items (25 items) | 0-1,201 | 0.0000-51.8300 | 2-282 |

**Panel C. Questionnaire completion time (N = 2,317)**

| **Statistic** | **Value** |
| --- | --- |
| Records (n) | 2,317 |
| Minimum (s) | 72 |
| 25th percentile (s) | 268 |
| Median (s) | 360 |
| 75th percentile (s) | 510 |
| Maximum (s) | 12,560 |
| Mean (SD) (s) | 476.7 (588.4) |
| Completion time <120 s, n (%) | 33 (1.42%) |
| Completion time <300 s, n (%) | 771 (33.28%) |

*Note: Panel A summarizes the data-cleaning rules; Panel B reports variables with missing or special values, including blank entries and questionnaire skip markers; Panel C summarizes the questionnaire completion time. The high missing percentages for the section-header variables reflect questionnaire logic and do not indicate item non-response. Implausible age values are present values and are therefore not counted as missing in Panel B. HPV, human papillomavirus.*

**Supplementary Table S3. Collinearity and model-fit diagnostics for the primary multivariable logistic regression model (n = 2,311)**

**Panel A. Collinearity diagnostics**

| **Predictor** | **VIF** | **Tolerance** |
| --- | --- | --- |
| Age | 1.9045 | 0.5251 |
| Rural residence | 1.0282 | 0.9725 |
| Married/divorced/widowed | 1.9523 | 0.5122 |
| Chronic disease | 1.0190 | 0.9813 |
| Family history of cervical disease | 1.0247 | 0.9759 |
| HPV vaccinated | 1.0623 | 0.9413 |
| Screening attitude score | 1.2170 | 0.8217 |
| Perceived susceptibility | 2.2910 | 0.4365 |
| Perceived severity | 2.3615 | 0.4235 |
| Perceived benefits | 1.8989 | 0.5266 |
| Perceived barriers | 1.9864 | 0.5034 |
| Cues to action | 2.1150 | 0.4728 |
| Self-efficacy | 2.2903 | 0.4366 |
| Health motivation | 2.6690 | 0.3747 |

**Panel B. Model-fit indices**

| **Statistic** | **Value** |
| --- | --- |
| Analytic N | 2,311 |
| −2 log-likelihood (final model) | 1,441.187 |
| Likelihood-ratio χ² (df) | 221.301 (14) |
| Likelihood-ratio p value | <0.001 |
| AIC | 1,471.187 |
| BIC | 1,557.368 |
| McFadden R² | 0.133 |
| Cox & Snell R² | 0.091 |
| Nagelkerke R² | 0.178 |

**Panel C. Box–Tidwell test of linearity of the logit for continuous predictors**

| **Predictor** | **x·ln(x) coefficient** | **z** | ***p* value** |
| --- | --- | --- | --- |
| Age | 0.428 | 1.081 | 0.280 |
| Screening-attitude score | 1.748 | 4.686 | <0.001 |
| Perceived susceptibility | −0.901 | −2.423 | 0.015 |
| Perceived severity | 0.042 | 0.113 | 0.910 |
| Perceived benefits | 0.371 | 1.107 | 0.268 |
| Perceived barriers | −0.551 | −1.367 | 0.172 |
| Cues to action | −0.025 | −0.063 | 0.950 |
| Self-efficacy | −0.761 | −1.891 | 0.059 |
| Health motivation | 0.286 | 0.672 | 0.502 |

*Note: Panel A reports variance inflation factor (VIF) and tolerance; all VIF values were below the conventional threshold of 10, indicating no substantial multicollinearity among predictors. Panel B reports model-fit indices for the same primary model. The education-restricted sensitivity analysis is reported in the main text (Table 6).* Panel C reports the Box–Tidwell test, which added a predictor × ln(predictor) term for each continuous predictor to the primary logistic model (n = 2,311); the likelihood-ratio test comparing the augmented and primary models was χ²(9) = 45.660, *p* < 0.001. No statistically significant departure from linearity was detected for perceived barriers, cues to action, perceived severity, or perceived benefits (all *p* > 0.15), whereas evidence of nonlinearity was observed for the screening-attitude score and perceived susceptibility; because the attitude score was included only as a covariate and perceived susceptibility was not independently associated with the outcome, continuous linear terms were retained in the primary model.

**Supplementary Table S4. Item-level reliability diagnostics for the Health Belief Model scale**

| **Domain** | **Item** | **Items in domain** | **Valid n** | **Mean** | **SD** | **Corrected item-total correlation** | **Alpha if item deleted** | **Domain alpha** |
| --- | --- | --- | --- | --- | --- | --- | --- | --- |
| Perceived susceptibility | q1 | 5 | 2317 | 2.0147 | 1.2788 | 0.8735 | 0.9396 | 0.9520 |
| Perceived susceptibility | q2 | 5 | 2317 | 1.9344 | 1.2513 | 0.9148 | 0.9325 | 0.9520 |
| Perceived susceptibility | q3 | 5 | 2317 | 2.0660 | 1.3196 | 0.8194 | 0.9496 | 0.9520 |
| Perceived susceptibility | q4 | 5 | 2317 | 1.7622 | 1.2314 | 0.8571 | 0.9425 | 0.9520 |
| Perceived susceptibility | q5 | 5 | 2317 | 1.7760 | 1.2007 | 0.8757 | 0.9395 | 0.9520 |
| Perceived severity | q6 | 5 | 2317 | 2.4514 | 1.4734 | 0.7401 | 0.9050 | 0.9153 |
| Perceived severity | q7 | 5 | 2317 | 2.7505 | 1.5541 | 0.8343 | 0.8855 | 0.9153 |
| Perceived severity | q8 | 5 | 2317 | 2.5559 | 1.5033 | 0.7909 | 0.8947 | 0.9153 |
| Perceived severity | q9 | 5 | 2317 | 2.9659 | 1.5363 | 0.8094 | 0.8909 | 0.9153 |
| Perceived severity | q10 | 5 | 2317 | 2.4005 | 1.3790 | 0.7434 | 0.9044 | 0.9153 |
| Perceived benefits | q11 | 4 | 2317 | 3.6711 | 1.5460 | 0.7730 | 0.8536 | 0.8901 |
| Perceived benefits | q12 | 4 | 2317 | 3.6448 | 1.5428 | 0.7970 | 0.8439 | 0.8901 |
| Perceived benefits | q13 | 4 | 2317 | 3.8433 | 1.3949 | 0.7195 | 0.8728 | 0.8901 |
| Perceived benefits | q14 | 4 | 2317 | 4.0047 | 1.3326 | 0.7531 | 0.8621 | 0.8901 |
| Perceived barriers | q15 | 7 | 2317 | 2.7907 | 1.4479 | 0.5899 | 0.9040 | 0.9032 |
| Perceived barriers | q16 | 7 | 2317 | 2.0561 | 1.3090 | 0.7534 | 0.8843 | 0.9032 |
| Perceived barriers | q17 | 7 | 2317 | 2.4178 | 1.3056 | 0.7736 | 0.8820 | 0.9032 |
| Perceived barriers | q18 | 7 | 2317 | 1.8312 | 1.2253 | 0.7809 | 0.8820 | 0.9032 |
| Perceived barriers | q19 | 7 | 2317 | 2.2451 | 1.3120 | 0.7610 | 0.8834 | 0.9032 |
| Perceived barriers | q20 | 7 | 2317 | 2.5662 | 1.4018 | 0.6727 | 0.8937 | 0.9032 |
| Perceived barriers | q21 | 7 | 2317 | 1.9404 | 1.3036 | 0.6851 | 0.8919 | 0.9032 |
| Cues to action | q22 | 3 | 2317 | 3.9318 | 1.3262 | 0.8613 | 0.9392 | 0.9454 |
| Cues to action | q23 | 3 | 2317 | 3.8817 | 1.3256 | 0.9112 | 0.9004 | 0.9454 |
| Cues to action | q24 | 3 | 2317 | 3.9629 | 1.2844 | 0.8857 | 0.9206 | 0.9454 |
| Self-efficacy | q25 | 3 | 2317 | 3.8235 | 1.2298 | 0.8006 | 0.8538 | 0.8984 |
| Self-efficacy | q26 | 3 | 2317 | 3.6677 | 1.2454 | 0.7874 | 0.8650 | 0.8984 |
| Self-efficacy | q27 | 3 | 2317 | 3.6888 | 1.2500 | 0.8094 | 0.8461 | 0.8984 |
| Health motivation | q28 | 6 | 2317 | 4.1597 | 1.1391 | 0.7947 | 0.9686 | 0.9651 |
| Health motivation | q29 | 6 | 2317 | 4.3940 | 1.0557 | 0.9248 | 0.9548 | 0.9651 |
| Health motivation | q30 | 6 | 2317 | 4.2956 | 1.0850 | 0.9391 | 0.9530 | 0.9651 |
| Health motivation | q31 | 6 | 2317 | 4.3643 | 1.0597 | 0.9523 | 0.9519 | 0.9651 |
| Health motivation | q32 | 6 | 2317 | 4.3293 | 1.0700 | 0.9489 | 0.9521 | 0.9651 |
| Health motivation | q33 | 6 | 2317 | 4.1152 | 1.1722 | 0.7875 | 0.9699 | 0.9651 |

*Note: Corrected item–domain total correlations were calculated within their corresponding domains. Alpha-if-item-deleted and domain alpha values were calculated using complete item sets within each domain. No total HBM composite score was computed or interpreted as a unidimensional measure.*

**Supplementary Table S5. Sampling adequacy and exploratory construct-validity results for the Health Belief Model scale**

**Panel A. Sampling adequacy**

| **Statistic** | **Value** |
| --- | --- |
| Kaiser-Meyer-Olkin (KMO) measure of sampling adequacy | 0.942 |
| Bartlett's test of sphericity | χ²(528) = 80,628.149, p < 0.001 |

**Panel B. Seven-component principal component analysis summary**

| **Component** | **Eigenvalue** | **Variance explained (%)** | **Cumulative (%)** |
| --- | --- | --- | --- |
| 1 | 11.1566 | 33.793 | 33.793 |
| 2 | 8.1479 | 24.680 | 58.473 |
| 3 | 2.3006 | 6.969 | 65.442 |
| 4 | 1.6459 | 4.985 | 70.427 |
| 5 | 1.2680 | 3.841 | 74.268 |
| 6 | 1.1142 | 3.375 | 77.643 |
| 7 | 0.8895 | 2.694 | 80.337 |

**Panel C. Varimax-rotated component loadings for the 33-item Health Belief Model scale (7 components requested)**

| **Item** | **Component 1** | **Component 2** | **Component 3** | **Component 4** | **Component 5** | **Component 6** | **Component 7** | **Communality** |
| --- | --- | --- | --- | --- | --- | --- | --- | --- |
| q1 | 0.248 | 0.001 | 0.071 | 0.276 | 0.059 | 0.833 | -0.001 | 0.839 |
| q2 | 0.235 | -0.020 | 0.063 | 0.289 | 0.043 | 0.863 | 0.015 | 0.889 |
| q3 | 0.270 | -0.003 | 0.073 | 0.218 | 0.025 | 0.809 | 0.015 | 0.780 |
| q4 | 0.180 | -0.037 | 0.017 | 0.346 | -0.011 | 0.822 | 0.067 | 0.833 |
| q5 | 0.241 | -0.049 | 0.025 | 0.323 | 0.015 | 0.827 | 0.056 | 0.853 |
| q6 | 0.657 | 0.059 | 0.104 | 0.216 | 0.078 | 0.447 | -0.042 | 0.701 |
| q7 | 0.799 | 0.078 | 0.146 | 0.215 | 0.095 | 0.295 | -0.053 | 0.811 |
| q8 | 0.759 | 0.069 | 0.123 | 0.210 | 0.058 | 0.313 | 0.040 | 0.744 |
| q9 | 0.823 | 0.079 | 0.234 | 0.229 | 0.093 | 0.152 | 0.047 | 0.826 |
| q10 | 0.646 | 0.004 | 0.140 | 0.367 | 0.038 | 0.359 | 0.074 | 0.707 |
| q11 | 0.451 | 0.177 | 0.714 | 0.059 | 0.160 | 0.059 | 0.074 | 0.782 |
| q12 | 0.394 | 0.164 | 0.749 | 0.064 | 0.154 | 0.073 | 0.102 | 0.787 |
| q13 | 0.012 | 0.307 | 0.797 | 0.138 | 0.170 | 0.075 | 0.095 | 0.792 |
| q14 | 0.099 | 0.379 | 0.766 | 0.116 | 0.201 | 0.034 | 0.080 | 0.802 |
| q15 | 0.240 | 0.061 | 0.321 | 0.646 | -0.050 | 0.097 | 0.005 | 0.594 |
| q16 | 0.067 | -0.061 | 0.088 | 0.766 | -0.091 | 0.333 | 0.099 | 0.732 |
| q17 | 0.227 | -0.007 | 0.105 | 0.793 | 0.068 | 0.191 | -0.043 | 0.734 |
| q18 | 0.067 | -0.128 | -0.026 | 0.741 | -0.044 | 0.457 | 0.112 | 0.794 |
| q19 | 0.220 | -0.012 | 0.010 | 0.751 | 0.114 | 0.283 | -0.039 | 0.708 |
| q20 | 0.333 | 0.037 | 0.075 | 0.693 | 0.228 | 0.144 | -0.148 | 0.694 |
| q21 | 0.077 | -0.093 | -0.091 | 0.664 | 0.031 | 0.425 | 0.171 | 0.674 |
| q22 | 0.142 | 0.368 | 0.242 | 0.093 | 0.801 | 0.032 | 0.127 | 0.881 |
| q23 | 0.121 | 0.387 | 0.221 | 0.059 | 0.803 | 0.052 | 0.214 | 0.910 |
| q24 | 0.110 | 0.426 | 0.249 | 0.040 | 0.751 | 0.045 | 0.256 | 0.889 |
| q25 | 0.008 | 0.525 | 0.135 | -0.021 | 0.276 | 0.098 | 0.669 | 0.828 |
| q26 | 0.027 | 0.444 | 0.096 | 0.080 | 0.215 | 0.044 | 0.736 | 0.803 |
| q27 | -0.001 | 0.467 | 0.141 | 0.035 | 0.147 | 0.066 | 0.760 | 0.842 |
| q28 | 0.138 | 0.718 | 0.192 | -0.020 | 0.306 | -0.022 | 0.355 | 0.791 |
| q29 | 0.087 | 0.915 | 0.168 | -0.033 | 0.178 | -0.068 | 0.076 | 0.917 |
| q30 | 0.053 | 0.918 | 0.174 | -0.041 | 0.159 | -0.036 | 0.143 | 0.924 |
| q31 | 0.085 | 0.933 | 0.175 | -0.032 | 0.171 | -0.057 | 0.088 | 0.951 |
| q32 | 0.057 | 0.938 | 0.158 | -0.040 | 0.137 | -0.036 | 0.113 | 0.942 |
| q33 | -0.054 | 0.827 | 0.091 | -0.031 | 0.059 | 0.033 | 0.238 | 0.758 |

*Note: Extraction method, principal component analysis; rotation, Varimax with Kaiser normalization. Loadings with an absolute value greater than 0.40 are treated as the primary loading of the item. Several items showed cross-loadings (absolute loadings >0.40 on more than one component): q6, q11, q18, q21, q24, q25, q26, and q27, consistent with the caution expressed in the main text that the seven-domain structure is preliminary. The Kaiser-Meyer-Olkin and Bartlett statistics indicated excellent sampling adequacy for principal component analysis. Seven components were requested to correspond to the theoretical HBM domains; the seventh component had an eigenvalue below 1.00 (Panel B). The Varimax-rotated solution (Panel C) showed a pattern broadly consistent with the hypothesized domains, but several items showed cross-loadings and the seventh component had an eigenvalue below 1.00. This analysis therefore provides only preliminary evidence and does not constitute validation of a seven-domain measurement model. Item-level content-validity indices and confirmatory factor analysis fit statistics were not available and are not reported; the proposed seven-domain structure should therefore be interpreted cautiously and confirmed in an independent sample.*

**Supplementary Figure S1. Participant and data-cleaning flow for the analytic sample**

*Note: A total of 2,317 questionnaire records were available for analysis. All records had complete data for personal screening willingness and the 33 Health Belief Model items. Six records contained invalid, non-numeric, header-like, or implausible age values and were retained for analyses not requiring age; age-specific analyses and the primary multivariable logistic regression therefore used 2,311 valid-age complete cases. No questionnaire records were excluded on* *the basis of completion time because no prespecified time-based exclusion threshold was available.*

**Supplementary Figure S2. Age and questionnaire completion-time distributions**

*Note: Panel A shows the distribution of the 2,311 valid numeric age observations; six records with invalid, non-numeric, header-like, or implausible age entries were treated as missing for age-specific analyses. Panel B shows the small age groups and the six invalid age entries. Panel C shows the distribution of questionnaire completion time for all 2,317 records (median 360 s; IQR 268-510 s; minimum 72 s; maximum 12,560 s). No records were excluded on the basis of completion time because no prespecified time-based exclusion threshold was available. The median age was 20 years (IQR 19-21 years), and 2,238 participants (96.8% of valid-age observations) were aged 18-24 years.*
